# An Obligatory Role of Mind Bomb-1 in Notch Signaling of Mammalian Development

**DOI:** 10.1371/journal.pone.0001221

**Published:** 2007-11-28

**Authors:** Bon-Kyoung Koo, Mi-Jeong Yoon, Ki-Jun Yoon, Sun-Kyoung Im, Yoon-Young Kim, Cheol-Hee Kim, Pann-Ghill Suh, Yuh Nung Jan, Young-Yun Kong

**Affiliations:** 1 Division of Molecular and Life Sciences, Pohang University of Science and Technology, Pohang, Kyungbuk, South Korea; 2 Department of Biology, Chungnam National University, Daejeon, South Korea; 3 Howard Hughes Medical Institute, Department of Physiology, University of California at San Francisco, San Francisco, California, United States of America; 4 Howard Hughes Medical Institute, Department of Biochemistry, University of California at San Francisco, San Francisco, California, United States of America; Ecole Normale Superieure, France

## Abstract

**Background:**

The Notch signaling pathway is an evolutionarily conserved intercellular signaling module essential for cell fate specification that requires endocytosis of Notch ligands. Structurally distinct E3 ubiquitin ligases, Neuralized (Neur) and Mind bomb (Mib), cooperatively regulate the endocytosis of Notch ligands in *Drosophila*. However, the respective roles of the mammalian E3 ubiquitin ligases, Neur1, Neur2, Mib1, and Mib2, in mammalian development are poorly understood.

**Methodology/Principal Findings:**

Through extensive use of mammalian genetics, here we show that *Neur1* and *Neur2* double mutants and *Mib2^−/−^* mice were viable and grossly normal. In contrast, conditional inactivation of *Mib1* in various tissues revealed the representative Notch phenotypes: defects of arterial specification as *deltalike4* mutants, abnormal cerebellum and skin development as *jagged1* conditional mutants, and *syndactylism* as *jagged2* mutants.

**Conclusions/Significance:**

Our data provide the first evidence that Mib1 is essential for Jagged as well as Deltalike ligand-mediated Notch signaling in mammalian development, while Neur1, Neur2, and Mib2 are dispensable.

## Introduction

The Notch signaling pathway is an evolutionarily conserved signaling module from nematode to human, which plays essential roles in pattern formation and cell fate determination through local cell-cell interactions [1]. Notch signaling is initiated by the interaction of the Notch receptors with their ligands, Delta [Deltalike (Dll) in mammals] and Serrate [Jagged (Jag) in mammals] [2], [3]. These interactions induce two sequential proteolytic cleavages of Notch receptor (S2 and S3 cleavages), and generate a soluble intracellular domain (Nicd) that translocates to the nucleus to form a transcriptional activator complex with Su(H)/CBF1/RBP-J. This complex activates the anti-neurogenic basic helix-loop-helix (bHLH) repressors.

Although much is known about Notch signal transduction after the receptor undergoes the ligand-dependent S2 cleavage, the mechanism by which the Notch ligands engage Notch receptors and trigger their cleavage is less understood. It has been suggested that the endocytosis of Notch ligands in the signal-sending cells is required for the effective activation of Notch signaling [4]. Two structurally distinct E3 ubiquitin ligases, Neuralized (Neur) and Mind bomb (Mib), regulate the endocytosis of the Notch ligand, Delta, in *Drosophila* and zebrafish, respectively [5]–[9]. Both proteins have been shown to interact with Delta and to promote its ubiquitination, endocytosis, and signaling. Interestingly, loss-of-function mutations in zebrafish *mib* and *Drosophila neur* cause similar expansions of the neural precursors, at the expense of the epidermis, through the loss of lateral inhibition [6], [9]. Since Mib and Neur share biochemical functions and their mutants show similar phenotypes, these two proteins were suggested to be functional homologues between species [10]. However, since the homologues of Mib and Neur are evolutionarily conserved, respectively, it has been suggested that a more complex interplay may exist between these E3 ubiquitin ligases and the endocytosis of Delta.

There are one *neur* (*dneur*) gene and two *mib* (*dmib1* and *dmib2*) homologue genes in *Drosophila*. Both dMib1 and dNeur are essential for a subset of the developmental events that are regulated by Notch signaling [10], [11]. *dmib1* mutants showed defects in wing margin specification, leg segmentation, and vein determination, while *dneur* mutants displayed increased numbers of neuroblasts and sensory organ precursors [9], [10]. Although dMib1 and dNeur regulate both Delta and Serrate [12], it has been suggested that dMib1 and dNeur are required for a distinct subset of Notch signaling events, primarily because of their differential expression patterns [10]. Interestingly, ectopic overexpression of dMib1 and dNeur in each other's mutants revealed their complementary functions, suggesting that they have similar molecular activities in *Drosophila* [10], [11]. Likewise, each mutant shows specific phenotypes, but also has a redundant phenotype, depending on the expression profiles. In fact, the *dneur* and *dmib* double mutant fly showed a complete block of lateral inhibition in sensory organ precursors [12]. Thus, in *Drosophila*, both the Delta and Serrate ligands require E3 ubiquitin ligases for their activity, and the requirement of dMib and dNeur appears to depend on the context.

In vertebrates, there are two Mib homologues, Mib1 and Mib2, and two Neur homologues, Neur1 and Neur2 [5], [6], [13]–[15]. All of them are implicated in the endocytosis of Notch ligands [5], [6], [13]–[15]. Mib1 is abundantly expressed in both embryos and adult tissues, while Mib2 is highly expressed in adult tissues, but only slightly in embryos [14]. Both Neur1 and Neur2 are expressed in the brain, but also specifically expressed in skeletal muscle and kidney, respectively [15]–[17]. Their differential expression profiles suggest that these E3 ligases may have redundant and/or specific functions in distinct contexts. Loss-of-function genetic studies revealed that unlike the *Drosophila neur* mutants, *Neur1^−/−^* mice have no defect in neurogenesis and embryonic development, possibly due to a functional overlap with *Neur2* [16], [17]. In contrast, *Mib1^−/−^* mice exhibited a neurogenic phenotype as well as pleiotropic Notch-related defects on embryonic day 9.5, suggesting the possible obligatory role of Mib1 in the regulation of multiple Dll and Jag ligands in mammalian development [13]. However, genetic evidence for these matters is still lacking, and other E3 ligases might have compensatory roles in the regulation of Notch ligands [14], [15]. To elucidate the interplay between the four E3 ubiquitin ligases and the Notch ligands in mammalian development, more comprehensive genetic studies of these four E3 ubiquitin ligases are required in a single organism.

There are five canonical Notch ligands, Dll1, Dll3, Dll4, Jag1, and Jag2 in mammals. Since Dll3 has no lysine residue for ubiquitin ligation [18], the others, Dll1, Dll4, Jag1, and Jag2, would be regulated by E3 ubiquitin ligases. The specific functions of each ligand have been identified through many genetic studies. Dll1 is required in somitogenesis and marginal zone B cell development, and Dll4 regulates arteriogenesis and angiogenesis [19]–[21]. *Jag1* conditional knockout mice revealed its role in cerebellum development and hair maintenance [22], [23]. *Jag2* mutant mice are well known to have *syndactylism* [24], [25]. These mutant studies have suggested the specific roles of each ligand in various Notch-mediated fate decisions. However, which E3 ligases are implicated in the distinct contexts regulated by each Notch ligand in mammalian development is largely unknown.

To establish the respective roles of these four E3 ubiquitin ligases in the various contexts of a single organism, we have generated *Neur2^−/−^*, *Neur1^−/−^*;*Neur2^−/−^* (*Neur1&2^DKO^*), *Mib2^−/−^*, and *Mib1* conditional mutant (*Mib1^f/f^*) mice. Both the *Neur2^−/−^* and *Mib2^−/−^* mice are viable, fertile, and grossly normal. Surprisingly, *Neur1&2^DKO^* mice are also viable and have no recognizable developmental defect, demonstrating the dispensable role of Neur1 and Neur2 in mammalian development. In contrast, the conditional inactivation of *Mib1* in the endothelium, skin epithelium, cerebellum, and apical ectodermal ridge faithfully showed the representative Notch phenotypes, such as the defects in arterial specification, and cerebellar and skin development exhibited by *Dll4*, and *Jag1* mutant mice, respectively, and the *syndactylism* shown in *Jag2^−/−^* mice. Our results clearly demonstrate the obligatory role of Mib1 in the regulation of Notch ligands during mammalian development.

## Results

### Generation of *Neur2^−/−^* mice

**Figure 1:**
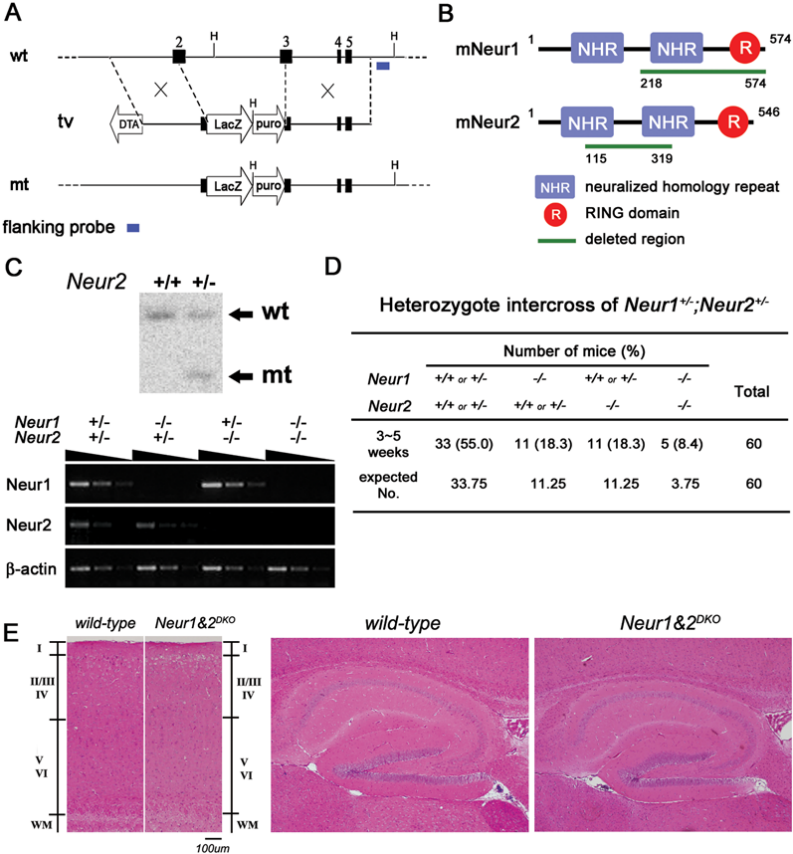
Generation of *Neur1* and *Neur2* double knockout mice and their dispensable role in mammalian cerebral development. (A) Targeted disruption of the murine *neuralized-2* (*Neur2*) locus. Schematic drawings of the *wild-type* (wt) and recombinant (mt) loci and the targeting vector (tv) are shown. The homologous recombination event deletes exons 2-3 and places the *IRES-LacZ* gene within the exon 2. H, *Hind*III. (B) Schematic drawing of the Neur1 and Neur2 proteins with the deleted region (green). (C) Southern blot analysis showing the recombination event. The RT-PCR analysis shows the loss of *Neur1* and *Neur2* transcripts in each mutant indicated. *β-actin* was used for normalization. wt, *wild-type* band; mt, *Neur2* mutant band. (D) Heterozygote intercrosses of *Neur1^+/−^;Neur2^+/−^* mice. (E) H&E sections of the neocortex (left panel) and hippocampus (right panel) of *wild-type* and *Neur1&2^DKO^* mice. Note that there is no difference between the *wild-type* and *Neur1&2^DKO^* mice.

The murine *Neur2* locus comprises 5 exons. The *IRES-lacZ-puro* cassette was fused to exon 2, with the deletion of exon 3 (Figure 1A). The deleted region encodes amino acids 115 to 319 of the murine Neur2 protein, which includes about 40% of the Neur2 protein (Figure 1B). This insertion abrogates the two neuralized homology repeats that are required for the interaction with Notch ligands [15], [26]. Based on our targeting strategy, *Neur2* transcripts will be fused with *IRES-lacZ* cDNA and lose the carboxy-terminal region of the Neur2 protein, which has the RING domain for ubiquitination (Figure 1A). After electroporation and drug selection, screening by Southern blotting with a 3′ external flanking probe using *Hind*III detected a homologous recombination event (Figure 1C). These clones were used to generate chimeric mice, and the heterozygous offspring were identified by PCR genotyping. When the heterozygous mice were intercrossed, the homozygous *Neur2^−/−^* offspring were viable and healthy. Using the primers specific for the undeleted 3′ region of the *Neur2* cDNA, these homozygous mice were confirmed as null for *Neur2* transcripts (Figure 1C). The homozygous mice were fertile and did not show any obvious Notch-related phenotypic abnormality up to now.

### Both Neur1 and Neur2 are dispensable for mammalian development

In *Drosophila*, there is one dNeur that is essentially required for Notch signaling in lateral inhibition [9]. In vertebrates, however, there are two Neur homologues, Neur1 and Neur2, which have similar degrees of homology to dNeur [15]. The mutant mice with a disruption in either *Neur1* or *Neur2* were healthy and had few Notch-related phenotypes [16], [17], suggesting possible compensation by each other. To test this possibility, we generated *Neur1&2^DKO^* mice. Surprisingly and unexpectedly, the *Neur1&2^DKO^* mice were born at the expected Mendelian ratios from intercrosses of *Neur1&2* double heterozygous mice (Figure 1D). These double mutant mice were confirmed by the absence of both *Neur1* and *Neur2* transcripts in the adult brains (Figure 1C). Microscopic analyses of histologic tissue sections revealed that there were no observable abnormalities in various tissues, including the brain (Figure 1E, not shown). Interestingly, *Neur1&2^DKO^* mice exhibited normal morphology in the cerebral cortex and hippocampal region, demonstrating that both Neur1 and Neur2 are dispensable for mammalian cerebral development. Taken together, all of mouse Neur homologues are dispensable for many developmental processes that are regulated by Notch signaling, such as somitogenesis, neurogenesis, vasculogenesis and limb and skin development.

### Mib2 is dispensable in mammalian development

**Figure 2:**
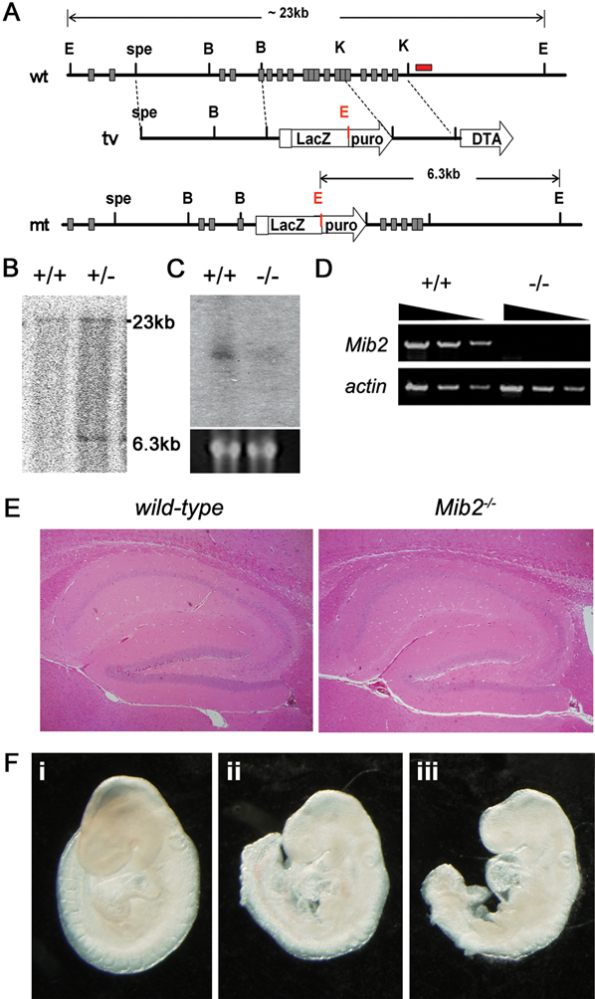
Generation of *Mib2^−/−^* mice and its dispensable role for mammalian development. (A) Gene targeting of the murine *Mib2* locus. The homologous recombination event deletes exons 5–15 and places the *IRES-LacZ* gene within exon 5. Schematic structures of the *wild-type* (wt) and recombinant loci (mt) and the targeting vector (tv) are shown. E, *EcoR*I; B, *BamH*I; K, *Kpn*I; spe, *Spe*I. (B) Southern blot analysis of tail DNA after *EcoR*I digestion with the flanking probe shown in (A). The positions of the *wild-type* (23kb) and the targeted (6.3kb) allele are indicated. (C) Northern blot analysis of *Mib2* gene expression in adult brain mRNA from *wild-type* (*+/+*) and *Mib2^−/−^* (*−/−*) mice. Loading and integrity of the RNA were assessed by ethidium bromide staining of the *28S* RNA in the gel prior to membrane transfer. (D) RT-PCR analysis of adult brain using *Mib2*-specific primers. No PCR product was detected from *Mib2^−/−^* cDNA. *β-actin* was used for the normalization. (E) H&E sections of the hippocampus of *wild-type* (left) and *Mib2^−/−^* (right) mice. Note that there is no difference between the *wild-type* and *Mib2^−/−^* mice. (F) Whole-mount images of E9.5 embryos. *Mib1^+/−^;Mib2^−/−^* (i), *Mib1^−/−^;Mib2^+/−^* (ii), and *Mib1^−/−^;Mib2^−/−^* (iii).

Both Mib1 and Mib2 have similar biochemical activity to Notch ligand(s), and Mib2 can rescue the Notch phenotypes of zebrafish *mib1* mutants [14]. In addition, *Mib2* is highly expressed in adult tissues, but only slightly in embryos [14]. Thus, we speculated that *Mib2* might function in adulthood, whereas *Mib1* has an obligatory role in embryos. To test this possibility, we generated *Mib2^−/−^* mice. The murine *Mib2* locus comprises 21 exons. The *IRES-lacZ-puro* cassette was inserted within exon 5, and the remaining region from exon 5 to exon 15 was replaced with the *IRES-lacZ-puro* cassette (Figure 2A). The deleted region encodes amino acids 152 to 656 of the murine Mib2 protein, encoding the Mib domain, the Mib repeat and the ankyrin repeat domain [14]. The deleted region includes over 50% of the Mib2 protein. The homozygous mutant (*Mib2^−/−^*) mice were generated as described for the *Neur2^−/−^* mice. Disruption of the *Mib2* gene was confirmed by northern blot and RT-PCR analyses of brain tissues (Figure 2C, D).

Heterozygous intercrosses produced their offspring at the predicted Mendelian ratio, and the *Mib2^−/−^* mice did not show any recognizable differences in growth, body weight, and health up to 12 months, as compared to the control littermates. In addition, the mutant brains in embryos as well as in adults were indistinguishable in both size and appearance from their control littermates (not shown). A histological analysis of the *Mib2^−/−^* brains revealed no detectable abnormalities at both the embryonic and adult stages (Figure 2E, not shown). These results demonstrate that Mib2 is not essential for Notch signaling-dependent developmental processes, including somitogenesis, neurogenesis, vasculogenesis and limb and skin development.

To reveal the effects of the loss of Mib2 in more sensitized condition, we crossed *Mib1^+/−^* mice with *Mib2^−/−^* mice to generate *Mib1^+/−^;Mib2^−/−^* and *Mib1^−/−^;Mib2^−/−^* mice. Although *Mib1^+/−^;Mib2^−/−^* mice have only one Mib homologue, these mice are also viable and have no recognizable abnormalities, suggesting that a single allele of *Mib1* gene is enough to regulate the mammalian Notch signaling (not shown). However, *Mib1^−/−^;Mib2^−/−^* mice showed embryonic lethality with similar defects with *Mib1^−/−^;Mib2^+/−^* or *Mib1^−/−^* mice (Figure 2F). Because the level of *Mib2* transcripts are very low compared to *Mib1* in the embryonic stages, this embryonic lethality are mainly caused by the loss of *Mib1* not *Mib2* [14]. Taken together, *Mib1* is the dominant *Mib* homologue that regulates the mammalian Notch signaling, while *Mib2* is dispensable.

### Generation of *Mib1* conditional knockout mice

Among the four E3 ubiquitin ligases for Notch ligands in mammals, only the *Mib1^−/−^* mice showed severe Notch-related phenotypes and early embryonic lethality (∼E10.5) [13], while the *Neur1&2^DKO^* and *Mib2^−/−^* mice did not show any obvious Notch-related phenotype until adulthood. Notch signaling is implicated in various cell fate decisions, not only in early embryogenesis but also in late organogenesis and the postnatal stages. Moreover, genetic studies of Notch ligands revealed the specific roles of each ligand in various Notch-mediated fate decisions. However, it is not known which E3 ligase is involved in these Notch signaling events. To clarify this issue, we generated *Mib1* conditional knockout mice carrying two *loxP* sites between exons 1 and 2 and between exons 3 and 4 (Figure S1). The homozygous mice (*Mib1^f/f^*) were generated as described for the *Neur2^−/−^* mice. The *Mib1^f/f^* mice were fully fertile and had no abnormalities. Moreover, *Mib1^f/−^* mice also had no abnormalities. To verify the generation of *Mib1^f/f^* mice, the null mice (*Mib1^Δ/Δ^*) were generated using *protamine-cre* transgenic mice that produce a germ line deletion of the *loxP*-floxed region. These *Mib1* ^Δ/Δ^ mice showed the same phenotypes as the *Mib1^−/−^* mice (not shown) [13], indicating that the *Mib1* conditional allele is fully functional and became a null allele by the Cre recombinase.

### Conditional inactivation of *Mib1* in endothelial cells generates a *Dll4* mutant phenotype

**Figure 3:**
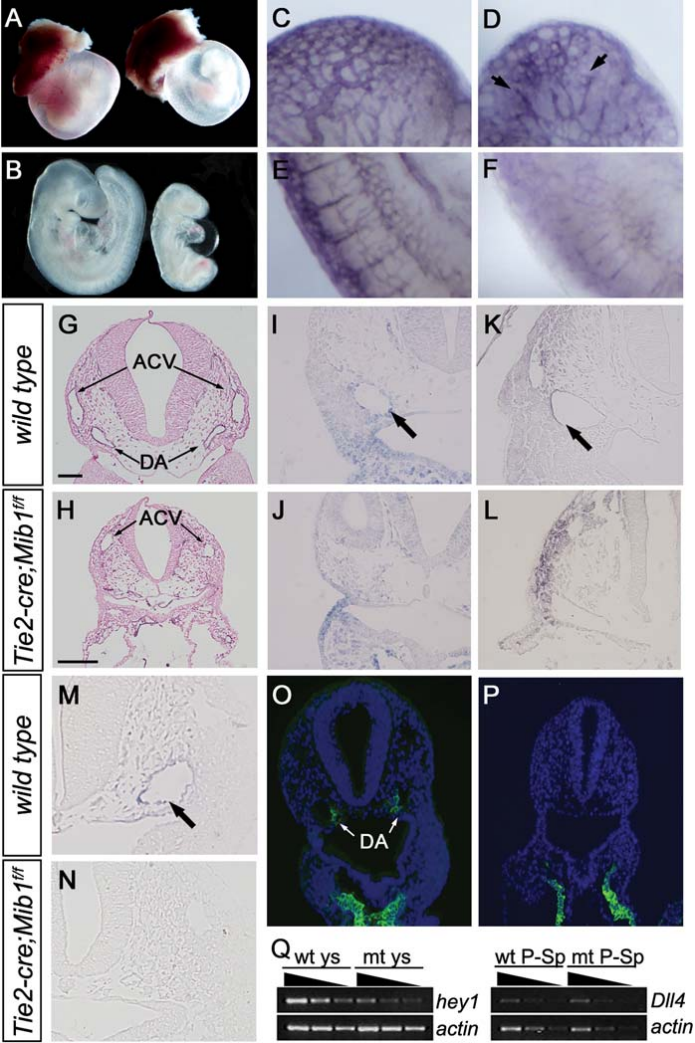
Conditional inactivation of *Mib1* in endothelial cells generates a *Dll4* mutant phenotype. (A, B) External view of embryonic day 9.5 (E9.5) yolk sacs (A) and embryos (B). The *Tie2-cre;Mib1^f/f^* yolk sac (A, right) has failed to remodel the primary vascular plexus to form large vitelline blood vessels. The *Tie2-cre;Mib1^f/f^* embryo (B, right) exhibits growth retardation and pericardial effusion. (C–F) Whole-mount PECAM staining of E9.5 *wild-type* (C, E) and *Tie2-cre;Mib1^f/f^* (D, F) embryos. The large cranial vessels appear truncated and degenerated in the mutants (D, arrows). Intersomitic vessels are present, but the angiogenic sprouts are disorganized in the mutants (F). (G, H) Sections of PECAM-1 stained embryos. The dorsal aortas of *Tie2-cre;Mib1^f/f^* embryos are reduced in diameter (H). ACV, anterior cardinal vein; DA, dorsal aorta. Scale bar, 200 µm. (I–L) Sections of *ephrinB2* (I,J) and CD44 (K,L) stained embryos. The dorsal aortas of *Tie2-cre;Mib1^f/f^* (J,L) embryos are negative for the arterial markers, *ephrinB2* and CD44. (M–P) Sections of *sm22* (M,N) and αSMA (O,P) stained embryos. The smooth muscle cell markers, *sm22* and αSMA, are lost in the dorsal aortas of *Tie2-cre;Mib1^f/f^* embryos (N,P). (Q) Semiquantitative RT-PCR in the yolk sac (ys) and para-aortic splenchnopleura (P-Sp) of *wild-type* (wt) and *Tie2-cre;Mib1^f/f^* (mt). *Hey1* is down-regulated in the mt yolk sac (left panel). *Dll4* expression in the mt P-Sp is similar to that of wt (right panel). *β-actin* was used for normalization.

Dll4 is responsible for arterial cell specification in vascular development [19], [20], [27]. Mib1 interacts with Dll4 and induces its endocytosis [13]. Therefore, we examined whether Mib1 is required for vascular development. The *Mib1^f/f^* mice were bred with *Tie2-cre* transgenic mice, which express Cre recombinase under the control of the *Tie2* promoter [28]. The *Tie2-cre;Mib1^f/f^* mice were embryonic lethal around E11.5. Therefore, the *Tie2-cre;Mib1^f/f^* embryos were dissected at E7.5-E10.5. At E8.5, there were no obvious differences between the *wild-type* and *Tie2-cre;Mib1^f/f^* embryos (not shown). Between E9.5 and E10.5, however, the *Tie2-cre;Mib1^f/f^* embryos exhibited characteristic vascular remodeling defects, such as a mottled avascular yolk sac, growth retardation, and pericardial effusion (Figure 3A, B). Staining of endothelial cells with an anti-PECAM1 antibody demonstrated the presence of a vessel structure consisting of endothelial cells in *Tie2-cre;Mib1^f/f^* embryos, indicative of intact vasculogenesis (Figure 3D, F). However, the *Tie2-cre;Mib1^f/f^* mice displayed a complete absence of vascular remodeling. The capillary network was less extensive and more primitive than that of the *wild-type* littermates (Figure 3D, F). Transverse sections of PECAM stained embryos revealed that the paired dorsal aorta in the *Tie2-cre;Mib1* ^f/f^ embryos were frequently narrower (Figure 3H). The observed mutant phenotypes were reminiscent of the *Dll4^+/−^* and *Dll4^−/−^* mice, suggesting that Mib1 regulates Dll4 in endothelial cells.

We further examined whether arterial cell fate is also impaired in *Tie2-cre;Mib1^f/f^* embryos. EphrinB2 is one of the earliest arterial-specific endothelial cell markers [29]. In *wild-type* embryos, e*phrinB2* was normally expressed in arteries, but not in *Tie2-cre;Mib1^f/f^* embryos, whereas its expression was retained in other tissues (Figure 3J). In addition, CD44 expression in the dorsal aorta, another arterial marker [30], was down-regulated in *Tie2-cre;Mib1^f/f^* embryos (Figure 3L). When the recruitment of smooth muscle cells to nascent vessel walls was examined, the expression of both *sm22* mRNA and α-smooth muscle actin protein (αSMA) were almost completely absent in the dorsal aorta of the *Tie2-cre;Mib1^f/f^* embryos (Figure 3N, P). Finally, the transcript of *hey1*, one of the Notch target genes responsible for arterial cell specification, was down-regulated in the *Tie2-cre;Mib1^f/f^* yolk sac. However, the *Dll4* transcripts in the *Tie2-cre;Mib1^f/f^* para-aortic splanchnopleura (P-Sp) were similar to those of the *wild-type* (Figure 3Q). These results demonstrate that Mib1 is required for arterial specification, which might be due to defective Dll4 activity.

### Conditional inactivation of *Mib1* in skin epithelial cells generates a *Jag1* mutant phenotype

**Figure 4:**
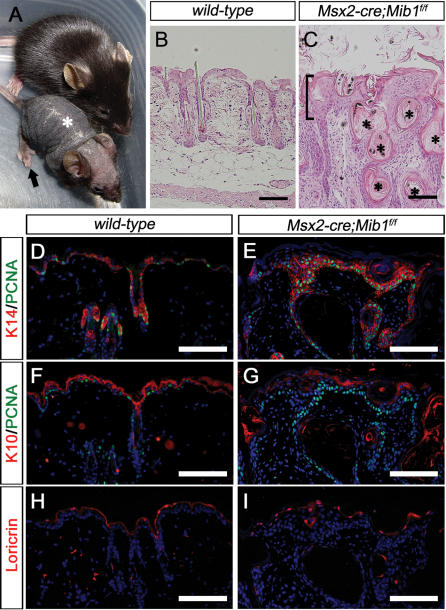
Conditional inactivation of *Mib1* in skin epithelial cells generates a *Jag1* mutant phenotype. (A) External view of postnatal day 28 (P28) mice. The *Msx2-cre;Mib1^f/f^* (lower) mouse shows loss of hairs in the dorsal midline (asterisk) and fused digits (arrow). (B, C) H&E sections of P28 *wild-type* (B) and *Msx2-cre;Mib1^f/f^* (C) skin. The *Msx2-cre;Mib1^f/f^* skin shows hyperplasia of skin epithelial cells (bracket) with lots of cysts (asterisks). Scale bar, 100 µm. (D–I) Sections of *wild-type* (D, F, H) and *Msx2-cre;Mib1^f/f^* (E, G, I) skin stained with K14/PCNA (D, E), K10/PCNA (F, G) and Loricrin (H, I). The *Msx2-cre;Mib1^f/f^* skin shows basal cell proliferation. K14, basal cell marker; K10, spinous cell marker; loricrin, granular cell marker; PCNA, proliferating cell marker. Scale bar, 100 µm.

Notch signaling plays important roles in epidermal differentiation and hair follicle maintenance [31], [32]. *Jag1* is expressed in the differentiated interfollicular epidermis (IFE), outer root sheath (ORS), pre-cortex, and matrix of hair follicles, and a genetic study revealed that Jag1 is the Notch ligand responsible for the maintenance of hair [22]. Since *Mib1* is also expressed in IFE and hair follicles and regulates the endocytosis of Jag1 [13], [14], we examined whether conditional inactivation of *Mib1* in the skin also reproduces the *Jag1* mutant phenotype in the skin. We bred *Mib1^f/f^* mice with *Msx2-cre* transgenic mice, in which the Cre recombinase is active not only in the apical ectodermal ridge at E11.5 but also in the dorsal ectoderm of early embryos and the matrix of hair follicles after birth [31], [33]. The *Msx2-cre;Mib1^f/f^* mice were smaller than their littermate controls and progressively lost their hair from the dorsal midline. They became completely bald at about 4 weeks after birth (Figure 4A).

At postnatal day 28 (P28), the back skin of the *wild-type* contained hair follicles in an anagen state (Figure 4B). In contrast, the hair follicles in the *Msx2-cre;Mib1^f/f^* back skin were barely detected, and large epidermal cysts below the interfollicular epidermis were formed as the hairs were progressively lost (Figure 4C), which is a representative phenotype of *Jag1* mutant mice [22]. In the *Msx2-cre;Mib1^f/f^* mice, the epidermal cell layers were thickened and lots of squames were observed (Figure 4C). In order to characterize the phenotype further, we examined the expression of various epidermal markers, such as undifferentiation (K14) and differentiation markers (K10, Loricrin), with proliferation marker (PCNA). The numbers of K14-positive basal or ORS cells were markedly increased in the *Msx2-cre;Mib1^f/f^* skin, as compared to the *wild-type* (Figure 4E), whereas the numbers of differentiated epidermal cells, such as K10- or Loricrin-positive cells, were reduced (Figure 4G, I). Most of the K14-positive cells were PCNA-positive, indicating that they are proliferating (Figure 4E). The enlarged cysts observed in the *Msx2-cre;Mib1^f/f^* skin were filled with cornified materials (Figure 4C), reminiscent of the enlarged cysts in other Notch-related mutants, such as *Notch1*, *Notch1&2*, *RBPjκ*, *presenilin1&2*, or *Jag1* deficient mice [22], [31], [32]. Taken together, the *Msx2-cre:Mib1^f/f^* mice showed peculiar skin epithelial phenotypes as in other Notch mutants, demonstrating that Mib1 is an essential E3 ubiquitin ligase that regulates epidermal differentiation and hair follicle maintenance.

### Conditional inactivation of *Mib1* in cerebellum generates a *Jag1* mutant phenotype

**Figure 5:**
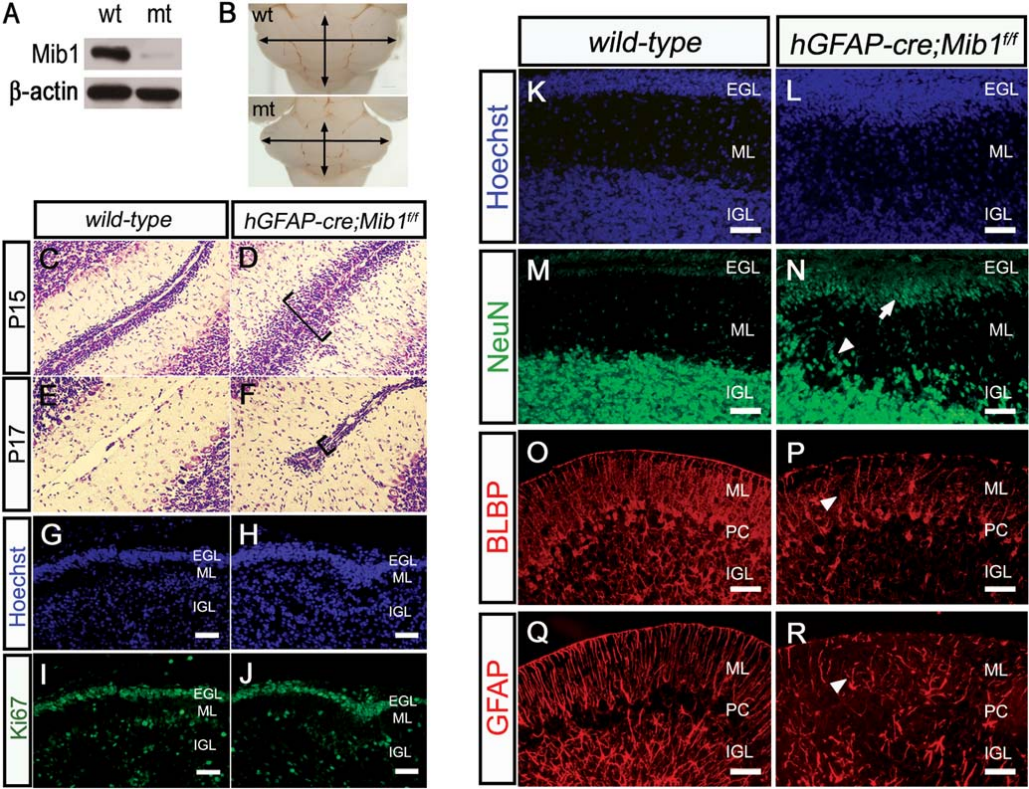
Conditional inactivation of *Mib1* in cerebellum generates a *Jag1* mutant phenotype. (A, B) Western blot analysis of P15 vermis (A) and whole-mount images of P9 vermis (B). wt, *wild-type*; mt, *hGFAP-cre;Mib1^f/f^*. β-actin was used for normalization. (C–F) Nissle stained sections of P15 (C, D) and P17 (E, F) vermis from *wild-type* (C, E) and *hGFAP-cre;Mib1^f/f^* (D, F) mice. *hGFAP-cre;Mib1^f/f^* sections show the accumulation of granule cells (D, F, bracket). (G–J) DNA staining by Hoechst (G, H) and Ki67 immunostaining (I, J) of *wild-type* (G, I) and *hGFAP-cre;Mib1^f/f^* (H, J) sections of P4 mice. Note that the *hGFAP-cre;Mib1^f/f^* EGL has a similar level of proliferation as compared to the *wild-type* EGL. (K–N) DNA staining by Hoechst (K, L) and NeuN immunostaining (M, N) of *wild-type* (K, M) and *hGFAP-cre;Mib1^f/f^* (L, N) sections of P15 mice. Note that the *hGFAP-cre;Mib1^f/f^* mouse shows delayed migration of postmitotic granule cells in EGL (arrow) and ML (arrowhead). (O–R) BLBP (O, P) and GFAP (Q, R) immunostaining of *wild-type* (O, Q) and *hGFAP-cre;Mib1^f/f^* (P, R) sections of P15 mice. The *hGFAP-cre;Mib1^f/f^* mouse shows truncated Bergmann glial fibers that fail to extend to the pial surface (P, R, arrowhead). EGL, external germinal layer; ML, molecular layer; IGL, internal granule cell layer; PC, Purkinje cell layer. Scale bars, 50 µm.

Previous analyses of *Notch1*, *Numb* and *Jag1* conditional mutant mice revealed that Notch signaling is important in cerebellar development [23], [34], [35]. Loss of *Jag1* results in delayed granule cell migration, potentially because of the mis-organized Bergmann glial fibers [23]. In order to examine whether Mib1 is required for cerebellar development, we bred *Mib1^f/f^* mice with *hGFAP-cre* transgenic mice, in which Cre recombinase is expressed in the Bergmann glia and granule cells, but not in the Purkinje cells, in the cerebellum [36]. A western blot analysis of a cerebellar lysate confirmed the ablation of *Mib1* in these mice (Figure 5A).

The *hGFAP-cre;Mib1^f/f^* mice were smaller than the *wild-type* littermates, and died between 2 to 4 months. While the cerebellum sizes were indistinguishable at P4, reductions in both the cerebellum size and foliation were recognizable in the mutant mice from P9 (Figure 5B). A histological analysis of the P15 *hGFAP-cre;Mib1^f/f^* cerebellum revealed the accumulation of granule cells in the external germinal layer (EGL), as compared to the *wild-type* (Figure 5C, D). At P17, granule cells in the EGL were still retained in the mutant cerebellum, but not in the *wild-type* (Figure 5E, F). At P21, however, all of the granule cells had finally migrated into the internal granule cell layer (IGL) in the mutant mice (not shown). These results suggest that granule cell migration is delayed in *hGFAP-cre;Mib1^f/f^* mice. An immunohistochemical analysis using Ki67, a proliferation marker, revealed a similar level of proliferation in the EGL at P4 (Figure 5I, J), suggesting that the accumulation of granule cells in the mutants is not caused by the increased proliferation. Consistent with this finding, an immunohistochemical analysis using a postmitotic neuronal marker, NeuN [37], showed the delayed migration of the postmitotic granule cell precursors from the EGL to the internal granule cell layer (IGL) in *hGFAP-cre;Mib1^f/f^* mice (Figure 5N).

A previous study suggested that the delayed migration of granule cells in the *En2-cre;Jag1^f/f^* conditional knockout cerebellum might be due to the mis-organized Bergmann glial fibers [23]. When we examined the integrity of the Bergmann glial fiber in the developing cerebellum with the glial markers, GFAP and BLBP, the Bergmann glial processes were severely disorganized in the P15 *hGFAP-cre;Mib1^f/f^* cerebellum, while the *wild-type* cerebellum showed radially organized processes of the Bergmann glia (Figure 5O–R). Taken together, the *hGFAP-cre;Mib1^f/f^* mice displayed the exact phenocopy of the *Jag1* conditional knockout mice, suggesting that Mib1 is an essential E3 ubiquitin ligase for Jag1 in the developing cerebellum.

### Conditional inactivation of *Mib1* in the apical ectodermal ridge generates a *Jag2* mutant phenotype

**Figure 6:**
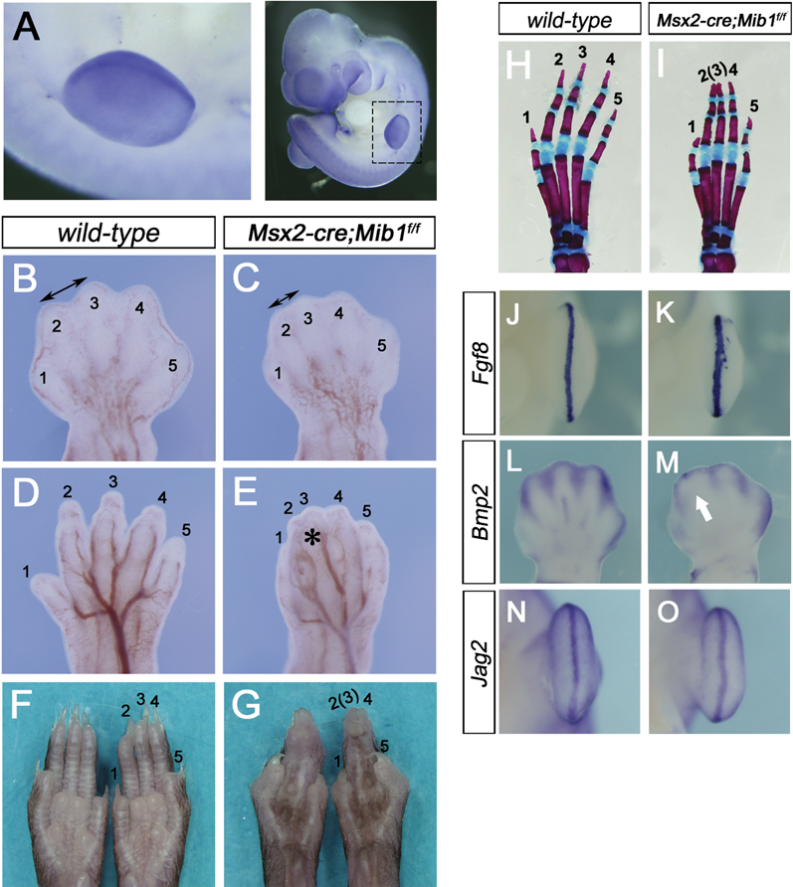
Conditional inactivation of *Mib1* in apical ectodermal ridge generates a *Jag2* mutant phenotype. (A) Whole-mount *in situ* hybridization of *Mib1* in E10.5 apical ectodermal ridge (AER). The image on the left is an enlarged view of the boxed area in the right image. (B–G) Hind limbs of E13.5 (B, C), E15.5 (D, E), and P21 (F, G) of *wild-type* (B, D, F) and *Msx2-cre;Mib1^f/f^* (C, E, G) mice. Note that the mutants show the fusion of digits 2 and 3 (C, E, G, asterisk). Numbers indicate the digit identity. (H, I) Stained skeletal preparations of neonatal hind limbs of *wild-type* (H) and *Msx2-cre;Mib1^f/f^* (I) mice. Numbers indicate the digit identity. (J–O) Whole-mount *in situ* hybridization of *Fgf8* (J, K, E11.5), *Bmp2* (L, M, E13.5), and *Jag2* (N, O, E11.5) in *wild-type* (J, L, N) and *Msx2-cre;Mib1^f/f^* (K, M, O) embryonic hind limbs. *Msx2-cre;Mib1^f/f^* embryos have broad *Fgf8* expression (K) and show the loss of interdigital *Bmp2* expression (M, arrow).

Syndactyly (digit fusions) is a well known *Jag2* mutant phenotype [24], [25]. Especially, the loss of Notch signaling in the apical ectodermal ridge (AER) of the embryonic limb bud causes this phenotype [38]. *Jag2* is expressed in the AER from E10.5, and its mutant mice show severe *syndactylism* that is equivalent to the phenotypes of *Notch1&2^DKO^* and *presenilin1&2^DKO^* mice, suggesting the exclusive role of Jag2 in this process. Interestingly, *Mib1* is also highly expressed in the AER and in the underlying mesenchyme at E10.5 (Figure 6A). To examine whether Mib1 functions as an essential E3 ubiquitin ligase of Jag2 in the developing AER, we crossed *Mib1^f/f^* mice with *Msx2-cre* transgenic mice. At E13.5, the developing hind limb showed primary digit identity, with 5 digit tips in the *wild-type* embryos (Figure 6B). In contrast, *the Msx2-cre;Mib1^f/f^* embryos had a narrower interdigital space between digits 2 and 3 (Figure 6C). At E15.5, the *Msx2-cre;Mib1^f/f^* mice had no digit separation (Figure 6E). Eventually, the *Msx2-cre*;*Mib1^f/f^* mice had fused digits (2∼4) in adulthood (Figure 6G). All of the mutant mice examined exhibited soft tissue fusions and fused nails of digits 2 and 3. Skeletal staining also revealed the fusion of the distal phalanges of digits 2 and 3 (Figure 6I).

To examine whether *Msx2-cre*;*Mib1^f/f^* embryos have similar molecular changes in the developing limb bud as in other Notch-related mutants, we analyzed the expression patterns of *Fgf8* (fibroblast growth factor 8; AER marker) and *Bmp2* (bone morphogenetic protein 2; interdigital cell marker at E13.5). Other notch-related mutant mice exhibit a hyperplastic AER and an expanded *Fgf8* expression domain [24], [25], [38]. As expected, the *Fgf8* expression domain in the newly formed AER of the hind limb bud was expanded in the *Msx2-cre*;*Mib1^f/f^* embryos, as compared to the *wild-type* (Figure 6J, K). Bmp signaling regulates apoptotic cell death in the interdigital regions, and Bmp expression is restricted to interdigital apoptotic cells [39], [40]. At E13.5, the *Msx2-cre;Mib1^f/f^* embryos exhibited reduced *Bmp2* expression in the interdigital region, especially between digits 2 and 3, suggesting decreased interdigital cell death (Figure 6M). Interestingly, the expression of *Jag2* in the AER of E11.5 embryos was not altered in the *msx2-cre*;*Mib1^f/f^* embryos, as compared to the *wild-type* (Figure 6N, O). This excludes the possibility that the loss of *Jag2* expression causes the syndactyly of the *Msx2-cre;Mib1^f/f^* mice. These data suggest that Mib1 functions as an essential E3 ubiquitin ligase of Jag2 that regulates the separation of digits in the developing limb.

## Discussion

Many E3 ubiquitin ligases are involved in regulating the Notch signaling pathway [41]. They are divided into two groups, one that ubiquitinates Notch receptors and the other that regulates Notch ligands. Two kinds of E3 ubiquitin ligases, Neur and Mib1, are important for the endocytosis of Notch ligands in *Drosophila* and zebrafish/mice, respectively [5]–[8], [13]. In *Drosophila*, depending on the developmental context, either dMib1 or dNeur is required for the activation of two Notch ligands, Delta and Serrate [10]–[12]. In mammals, there are two Mib homologues, Mib1 and Mib2, and two Neur homologues, Neur1 and Neur2, along with five Notch ligands, Dll1, Dll3, Dll4, Jag1, and Jag2, in the genome. However, the requirement of each E3 ubiquitin ligase in the developmental contexts where Notch signaling is active was largely unknown. In this report, we have analyzed the phenotypes of *Neur2^−/−^*, *Neur1&2^DKO^*, *Mib2^−/−^*, and *Mib1^f/f^* mice expressing the Cre enzyme by various promoters. Unexpectedly, only the *Mib1* conditional mutant mice faithfully exhibited the various Notch phenotypes representating each of the *Dll4*, *Jag1*, and *Jag2* mutant mice. Collectively, our extensive genetic studies reveal that Mib1 is the E3 ubiquitin ligase responsible for the regulation of Notch ligands in mammalian development.

### An obligatory role of Mib1 in mammalian development

In *Drosophila*, *dneur* mutants were initially identified by the loss of lateral inhibition in neurogenesis, which results in the overproduction of neuroblasts at the expense of the epidermoblast [9]. Although dMib1 and dNeur have similar functions and are exchangeable in some developmental contexts, the endogenous level of dNeur is important for the lateral inhibition that inhibits the neuronal fate in the epidermis [10], [11]. Moreover, the overexperssion of dMib1 in *dneur* mutant *Drosophila* cannot rescue the cuticular neurogenic phenotype of *dneur* mutant embryos, indicating a non-redundant function of Neur in this context [10]. Consistently, *dneur* is expressed throughout the ectoderm during neuronal cell fate specification [42]. Likewise, both murine *Neur1* and *Neur2*, which have a similar phylogenic distance from *dneur*, are expressed in the embryonic and adult brain [15]. In this study, however, the *Neur1&2^DKO^* mice did not show any obvious developmental defects in brain development and their other tissues were grossly normal, demonstrating that both Neur1 and Neur2 are dispensable in the neurogenesis where Notch-mediated lateral inhibition controls the number of neurons in mammalian development. Although we did not observe an obvious Notch phenotype in the *Neur1&2^DKO^* mice, we cannot exclude a possible role in the fine regulation of Notch ligands. Since *Neur1^−/−^* mice have olfactory discrimination defects and ethanol hypersensitivity [16], the *Neur1&2^DKO^* mice could have more severe defects in their behavior, although they did not exhibit a prominent developmental defect.

In contrast, we previously reported that *Mib1^−/−^* mice exhibit a severe neurogenic phenotype in the developing brain and neural tube in E9.5 embryos [13], demonstrating that Mib1 is essential for the lateral inhibition of embryonic neurogenesis, and the endogenous levels of both *Neur1* and *Neur2* cannot compensate for the loss of *Mib1*. In addition, the endocytosis of Dll1 was completely impaired in the *Mib1^−/−^* mice, suggesting that Mib1 is essentially required for Dll1 endocytosis [13]. Consistently, the overexpression of either mouse Neur1 or Neur2 alone does not promote the endocytosis of *Xenopus* Delta in Cos7 cells, while mouse Mib1 does [15]. Furthermore, zebrafish *mib1* mutants also showed DeltaD accumulation in the plasma membrane and a neurogenic phenotype that is a representative Notch defect of lateral inhibition in neurogenesis [6]. Consistent with these findings, the overexpression of mouse *Neur2* in the zebrafish *mib1* mutants could not reduce the increased number of *zath1*-positive hair cells, which results from the failure of lateral inhibition in the mechanosensory organs [15]. Abundant evidence suggested that Mib1, but not Neur1/2, might be the E3 ubiquitin ligase that regulates Notch ligands in mammalian development.

Previously reported *Mib1^−/−^* mice exhibited multiple defects in neurogenesis, somitogenesis, vasculogenesis, and cardiogenesis [13]. The developmental defects shown in *Mib1^−/−^* mice might be caused by the loss of Dll1 (neurogenesis, somitogenesis), Dll4, (vasculogenesis, cardiogenesis) signaling [13]. In this study, the *Tie2-cre;Mib1^f/f^* embryos also exhibited the vascular defects characteristic of *Dll4^+/−^* and *Dll4^−/−^* mice, such as defective remodeling and impaired arterial fate specification. Since the Dll4-Notch1 signaling pathway is essential for arterial fate specification, our data strongly suggest that Mib1 is the E3 ligase that regulates Dll4 activity in endothelial cells. Moreover, conditional inactivation of *Mib1* in hematopoietic system also exhibited the characteristic phenotype of *Dll1* mutants, the defect in the formation of marginal zone B cells (Song *et al*., our unpublished observation).

In addition to the regulation of Dll ligands, our data suggested that Mib1 is also the E3 ubiquitin ligase that regulates Jag ligands in mammalian development. The *hGFAP-cre;Mib1^f/f^* mice exhibited delayed granule cell migration in the cerebellum. In addition, the *Msx2-cre*;*Mib1^f/f^* mice showed hair follicle loss and epidermal cyst formation in the skin. These are both representative phenotypes of *Jag1* conditional knockout mice [22], [23]. Furthermore, the *Msx2-cre*;*Mib1^f/f^* mice also showed *syndactylism*, which is a well known Notch phenotype of *Jag2* null mice [24], [25]. Among the five canonical Notch ligands in mammals, Dll3 lacks a lysine residue in the cytoplasmic domain for ubiquitination, suggesting that it might not be a direct substrate of the E3 ubiquitin ligases. Except for Dll3, the other four Notch ligands, Dll1, Dll4, Jag1, and Jag2, should be regulated by E3 ligases for their endocytosis, because a plethora of evidence suggested that ligand endocytosis in the signal-sending cells is required for proper Notch activation in the signal-receiving cells, and that E3 ligases regulate their endocytosis. While *Neur1&2^DKO^* and *Mib2^−/−^* mice did not show any obvious Notch phenotype, the *Mib1^−/−^* mice in the previous study and the *Mib1* conditional knockout mice in various tissues in the present study clearly displayed the representative Notch phenotypes of each Notch ligand-null strain [13]. Thus, we concluded that Mib1, but not Neur1/2, plays an exclusive role in mammalian development, by potentially regulating the endocytosis of Jag ligands as well as Dll ligands.

### Evolutionary flux in the regulation of Notch ligands by E3 ligases

The Notch signaling pathway is evolutionarily conserved from nematode to human. In terms of the requirement of E3 ligase for Notch ligand endocytosis, however, evolutional divergence may exist. In *C. elegans*, in which the Notch signaling pathway is conserved as in *Drosophila* and vertebrates, there is a *neur* homologue, but no *mib* homologue. However, in contrast to the strict requirement of Notch ligand endocytosis in *Drosophila*, DSL ligand endocytosis does not seem to be conserved in *C. elegans*. The extracellular domains of DSL ligands can fully rescue the *lag-2* mutant and activate Notch signaling, while the intracellular domain-deleted mutants of Delta have a dominant-negative activity in *Drosophila* [43]–[45]. In zebrafish, *mib1* mutants accumulate DeltaD in the plasma membrane, and in a transplantation experiment, *mib1* null cells could not activate Notch signaling in the adjacent signal-receiving cells, indicating a strong requirement for ligand endocytosis in the Notch activation in zebrafish as well [6]. Consistently, *Mib1^−/−^* mice, in which the endocytosis of Dll1 was completely impaired and Dll1 accumulated in the plasma membrane, exhibit reduced expression of Notch target genes and loss of N1icd generation [13]. Furthermore, *Mib1^−/−^* cells cannot activate Notch signaling in a co-culture system with C2C12-Notch1 cells containing a CBF-Luciferase reporter gene [13]. Collectively, the requirement of E3 ligase in Notch ligand endocytosis appears to be well conserved from *Drosophila* to mammals.

In the regulation of Notch ligand endocytosis, however, two structurally distinct E3 ligases, Mib and Neur, might have evolved differently. In *Drosophila*, both Notch ligands, Delta and Serrate, require an E3 ubiquitin ligase, either dMib or dNeur, for their activity in Notch-mediated cell fate decisions dependent on expression patterns [10]–[12]. In mammals, however, Mib1 is essential for Notch-mediated cell fate decisions in all contexts of mammalian development examined in this study and the previous studies [13]. In contrast, the *Neur1^−/−^* mice generated by two independent groups did not show any apparent developmental Notch defects [16], [17]. In this study, the *Neur2^−/−^* and *Neur1&2^DKO^* mice also were grossly healthy without any observable abnormality. Consistent with this finding, Neur1 and Neur2 do not induce endocytosis of Notch ligands in various cells, including Cos7 cells [15], whereas Mib1 and Mib2 readily do [13], [14]. Although *Xenopus* Neur1 has been suggested to regulate Notch ligands in lateral inhibition, as revealed by an overexpression experiment [5], the loss-of-function studies using zebrafish *mib1* mutants and *Mib1^−/−^* and *Mib1^f/f^* mice indicated that Mib1 is required for various Notch-dependent cell fate decisions [6], [13]. Thus, the two structurally distinct E3 ligases, Mib and Neur, might have evolved differently in the Notch signaling pathway during evolution, especially in insects and vertebrates.

There are two Mib homologues, Mib1 and Mib2, from *Drosophila* to human [14]. As described above, Mib1 is functionally well conserved from *Drosophila* to mice. However, a substantial disparity exists, at least between zebrafish and mice. The zebrafish *mib1^ta52b^* mutants accumulated DeltaD in the plasma membrane and have various Notch mutant phenotypes, affecting neurogenesis, somitogenesis, vasculogenesis, and hematopoiesis. Interestingly, overexpression of Mib2, but not Neur1 and Neur2, rescues the neurogenic and vasculogenic defects in these mutants [14], [15], suggesting that Mib2 can regulate Notch ligands in these contexts. In fact, a recent comparative analysis of zebrafish *mib1* mutants with an antimorphic allele, *mib1^ta52b^*, and a null allele, *mib1^tfi91^* suggested that Mib1 and Mib2 might have a redundant role in Notch signaling [46]. The *mib1^tfi91^* mutants exhibited no obvious somite defects, while the *mib1^ta52b^* mutants have a severe somite phenotype, suggesting that the antimorphic *mib1^ta52b^* mutant with a mutation in the RING domain might suppress the Mib2 function in the *mib1* ^ta52b^ mutants, and that both Mib1 and Mib2 might function in somite formation. In contrast, two independently generated *Mib1^−/−^* mouse strains [13], [47] and the *Mib1* ^Δ/Δ^ mice conditionally deleted by the *protamine-cre* system in this study display defects in somitogenesis, with only a few anterior somites with irregular shapes. Thus, the dependency of Mib1 and Mib2 in species might have evolved differently.

We cannot completely exclude the possibility that other E3 ligases, Mib2, Neur1, and Neur2, might play a critical role in the fine regulation of Notch signaling, which could not be recognized in the present assay systems that have been developed in order to elucidate Notch contexts, because of technical limitations. However, the inactivation of Mib1 faithfully reproduced well-known Notch phenotypes, such as defects in neurogenesis, somitogenesis, vasculogenesis, hematopoiesis, skin morphogenesis, etc., in all contexts of mammalian development examined, without exception. Thus, our study strongly suggests that Mib1 is the core E3 ubiquitin ligase that regulates Notch ligands. This provides the first genetic evidence that Mib1 is required for the Notch-mediated cell fate decisions in a single organism, especially in mammals.

## Methods

### Mice

The *Mib1^f/f^*, *Mib2^−/−^*, and *Neur*2*^−/−^* mice were generated through the germ-line transmission of chimeric C57BL/6 mice with targeted E14K ES cells, and the *Neur1^−/−^* mice were previously generated [16]. The transgenic mouse lines, *Tie2-cre*, and *hGFAP-cre*, were purchased from Jackson Laboratories, and the *Msx2-cre* mouse line was kindly provided by B. Harfe and G.R. Martin. All of the mice were maintained in our animal colony under institutional guidelines.

### Southern and northern blot analyses and RT-PCR analysis

For the Southern blot analysis, genomic DNA from ES cells or mouse tail tip cells was digested by the appropriate restriction enzymes and fractionate by agarose gel electrophoresis. The DNA was transferred to a nylon membrane and analyzed with ^32^P-labeled DNA probes. For the northern blot analysis, freshly isolated RNA from mouse brain tissues was separated by agarose gel electrophoresis, transferred to a nylon membrane, and analyzed with ^32^P-labeled DNA probes. For the RT-PCR analysis, total RNA samples were extracted from mouse brains, using TRI-reagent (Sigma) according to the manufacturer's instructions. Aliquots of 1 or 2 μg RNA were used for reverse transcription (Omniscript RT, Qiagen) with oligo-dT priming. Primer information will be provided upon request.

### Western blot analysis

For the western blot analysis, equal amounts of tissue extracts were separated by SDS-PAGE and transferred to PVDF membranes. Membranes were incubated with antibodies to Mib1 (gift from Dr. P. Gallagher) and β-actin (Sigma). Protein bands were detected by enhanced chemiluminescence (Amersham Pharmacia Biotech).

### Histology and Immunohistochemistry

For histological analysis, tissues were fixed in 4% paraformaldehyde overnight at 4°C and embedded in paraffin wax or O.C.T. for sectioning. The sections were subjected to hematoxylin and eosin (H&E) or Nissle staining. For immunohistochemistry, paraffin-embedded or O.C.T.-embedded sections were used, and the antigenic epitopes were exposed using 10 mM citrate buffer and micro-waving. Sections were incubated in blocking solution (3% BSA, 5% goat serum or horse serum, and 0.5% Tween-20 in PBS) at RT for 4hr, followed by an additional incubation with various antibodies (Abs). Specific binding was detected with an ABC kit (Vectastain), an Envision kit (DAKO) or Alexa-488 (green) and/or -594 (red)-labeled Ab (Molecular Probes). Antibody probes included PECAM (1∶200, MEC13.3, BD), CD44 (1∶200, BD), αSMA (1∶200, NeoMarker), K14 (1∶1000, Covance), K10 (1∶500, Covance), Loricrin (1∶500, Covance), PCNA (1∶200, Santa Cruz Biotechnology), Ki67 (1∶200, Novocastra), NeuN (1∶100, Chemicon), BLBP (1∶2000, gift from Dr. N Heintz), and GFAP (1∶3000, Dako).

### *In situ* hybridization and bone staining

Details of the RNA *in situ* hybridizations on whole mount or sectioned embryos were described [13]. The *ephrinB2*, *sm22*, *Fgf8*, and *Jag2* DIG-labeled (digoxigenin) riboprobes were generated from pGEM-T vectors (Promega) containing amplified cDNA fragments (about 700∼800 bp). Staining patterns were confirmed by comparisons with previously published data. The *bmp2* probe was kindly provided by M. Logan. For the skeletal analysis, following removal of the skin, the specimen was fixed overnight in ethanol, and stained with Alcian blue and Alizarin red.

## Supporting Information

Figure S1 Generation of *Mib1* conditional knockout mice. (A) Schematic drawing of the targeting strategy. The *wild-type* allele (wt) was recombined with the conditional targeting vector (tv) to generate the floxed allele (floxed). As a result, one *loxP* sequence and a *loxP-neomycin* cassette are inserted between exons 1 and 2 and exons 3 and 4 of the mouse *Mib1* locus, respectively. Upon Cre expression, the floxed allele loses its exons 2 and 3 and becomes a null allele (deleted). Ev, *EcoR*V; H, *Hind*III; DTA, *Diphtheria toxin A*; neo, *neomycin* resistance gene. (B) The genomic Southern blot analyses by *EcoR*V (Ev) with a flanking probe and *Hind*III (H) with an internal probe show targeted embryonic stem cell clones (middle lane).
(8.74 MB TIF)
