# Supplementary figures and images for: An Obligatory Role of Mind Bomb-1 in Notch Signaling of Mammalian Development
Source: PLoS One. 2007 Nov 28;2(11):e1221. doi: 10.1371/journal.pone.0001221 (PMC2082076; doi:10.1371/journal.pone.0001221)

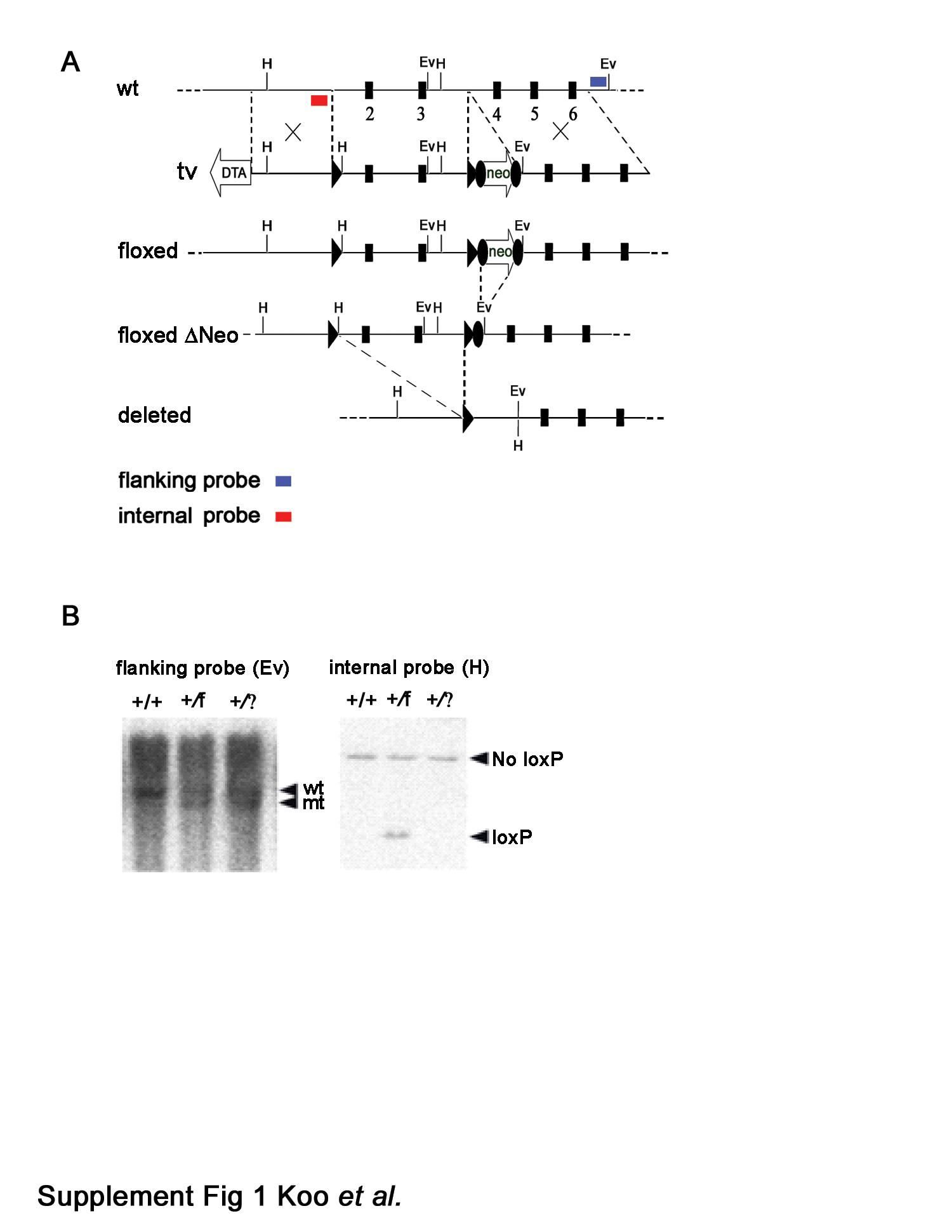

Supplement: Figure S1 — Generation of Mib1 conditional knockout mice. (A) Schematic drawing of the targeting strategy. The wild-type allele (wt) was recombined with the conditional targeting vector (tv) to generate the floxed allele (floxed). As a result, one loxP sequence and a loxP-neomycin cassette are inserted between exons 1 and 2 and exons 3 and 4 of the mouse Mib1 locus, respectively. Upon Cre expression, the floxed allele loses its exons 2 and 3 and becomes a null allele (deleted). Ev, EcoRV; H, HindIII; DTA, Diphtheria toxin A; neo, neomycin resistance gene. (B) The genomic Southern blot analyses by EcoRV (Ev) with a flanking probe and HindIII (H) with an internal probe show targeted embryonic stem cell clones (middle lane). (8.74 MB TIF) [file pone.0001221.s001.tif]
